# Cardiovascular Disease Risk Varies by Birth Month in Canines

**DOI:** 10.1038/s41598-018-25199-w

**Published:** 2018-05-17

**Authors:** Mary Regina Boland, Marc S. Kraus, Eddie Dziuk, Anna R. Gelzer

**Affiliations:** 1 Department of Biostatistics, Epidemiology and Informatics, Perelman School of Medicine, University of Pennsylvania, Philadelphia, Pennsylvania USA; 2 Institute for Biomedical Informatics, University of Pennsylvania, Philadelphia, Pennsylvania USA; 3 Center for Excellence in Environmental Toxicology, University of Pennsylvania, Philadelphia, Pennsylvania USA; 4 Department of Biomedical and Health Informatics, Children’s Hospital of Philadelphia, Philadelphia, Pennsylvania USA; 5 Department of Clinical Studies, School of Veterinary Medicine, University of Pennsylvania, Philadelphia, Pennsylvania USA; 6 Orthopedic Foundation for Animals, Columbia, Missouri USA

## Abstract

The canine heart is a robust physiological model for the human heart. Recently, birth month associations have been reported and replicated in humans using clinical health records. While animals respond readily to their environment in the wild, a systematic investigation of birth season dependencies among pets and specifically canines remains lacking. We obtained data from the Orthopedic Foundation of Animals on 129,778 canines representing 253 distinct breeds. Among canines that were not predisposed to cardiovascular disease, a clear birth season relationship is observed with peak risk occurring in June-August. Our findings indicate that acquired cardiovascular disease among canines, especially those that are not predisposed to cardiovascular disease, appears birth season dependent. The relative risk of cardiovascular disease for canines not predisposed to cardiovascular disease was as high as 1.47 among July pups. The overall adjusted odds ratio, when mixed breeds were excluded, for the birth season effect was 1.02 (95% CI: 1.002, 1.047, p = 0.032) after adjusting for breed and genetic cardiovascular predisposition effects. Studying birth season effects in model organisms can help to elucidate potential mechanisms behind the reported associations.

## Introduction

### Importance of Trans-Species Understanding of Disease

Trans-species polymorphisms are genetic polymorphisms shared across evolutionarily related species^1^. Five well-described ‘trans-species’ genes include the major histocompatibility complex (MHC), the ABO blood group, antiviral genes and auto-immune related genes^1^. Many trans-species gene studies were conducted in primates. However, canines (*Canis familiaris*) represent a particularly relevant animal model when studying human diseases because of their cohabitation with humans, which has resulted in similar environmental pressures^2^. For example, the FGF5 gene responsible for determining hair length in canines^3^ has also been implicated in baldness in humans^4^. Other mutations have been selected for in canine populations due to their desirability. For example, chondrodysplasia has been selected for in breeds such as the dachshund (literally ‘badger dog’ in German) that were breed for characteristics (such as short stature) that made them suitable for a specific purpose (such as hunting badgers that required an animal that was both short and long). This selection bias by design in domesticated canines has made mutations in FGF4 (chondrodysplasia gene) relatively common among short-legged canines^2,5^.

### Genetic Similarity Among Species with Strong Structural Similarity

Structural similarity is often used as a proxy for genetic similarity when detailed genetic information is unavailable with an estimated 20% of similar structures sharing a common genetic etiology^6^. In the cardiovascular domain, the canine cardiovascular system is a commonly used model for the human cardiovascular system because of similarities in physiology between canine and human hearts^7,8^. Further, breed-specific variance in risk for certain types of cardiovascular disease suggests a genetic contribution to disease risk among canines^9^.

### Role of Birth Month/Season in Canines

Recently, an association between birth season (using the proxy of birth month) and risk for cardiovascular disease has been uncovered in humans^10^ and replicated at other sites and regions^11,12^. Several hypotheses have been proposed in the literature, including increased perinatal influenza infection for those born in the winter in New York City or decreased perinatal vitamin D exposure^10,11^. Another explanation is first-trimester exposure to fine air particulates in humans, which is correlated with increased risk of atrial fibrillation later in life^13^. We expect the effect of seasonality on developing fetuses (either humans or canines) to be less drastic then pharmacological exposures^14^, however seasonal exposures still warrant further study^13^. Prior studies have established the presence of seasonal variance in animal births, which is well known and described^15,16^ the effect of birth season on disease risk in animals (especially those commonly used as animal models) is understudied. The majority of animal birth season research focuses on effects of food availability that varies with the season. Given that wild animals rely heavily on either foraging or hunting, seasonal variability in food supply effects the timing of their birth season and the survivability of offspring^17,18^.

The domesticated dog (*Canis familiaris*) differs widely from the wolf (*Canis lupus*) with regards to birth seasonality. Domesticated dogs’ are not subjected to the physical constraints of living in the wild^18^. In addition, domesticated dogs are bred for various purposes (e.g., working, enjoyment) that are human-centric and therefore their mating season is more tightly connected with human activities then their wild counterparts. Typically the wolf (*Canis lupus*) gives birth only in April and May in the Northern hemisphere (e.g., Alaska)^19^. However, domesticated dogs (*Canis familiaris*) breed throughout the year, which is believed to be due to regulation of temperature and food source stability^20^. Thus making the domesticated dog more similar to humans in that sexual reproduction is less tightly coupled to external climate changes and takes place throughout the year.

The purpose of this study is to investigate whether a relationship exists between birth season and cardiovascular disease risk among canines given the physiological similarities of canine and humanoid cardiovascular systems.

## Materials and Methods

### Dataset

The Orthopedic Foundation of Animals (OFA) has created an open database of dogs and cats (http://www.ofa.org/index.html) that contains certain key health information pertaining to breeding and overall health and wellness of animals. Health information consists of various types of reports including: radiographical evaluations, eyes, soft tissues (e.g., thyroid), other evaluations (e.g., temperament tests) and DNA test results (for known genetic diseases that afflict certain breeds). The majority of this information is accessible via an online browser. The dataset at the patient-level is not available for direct download, but could be viewed in the browser at the patient-level (http://www.offa.org/display.html?regnum=NP27924804#animal), but only for breeders who consent to allowing their dogs’ health information to be made public. Therefore, a parser was built to process the publically available information from the web interface and placing it into a computer-readable format.

The parser first obtains a list of all animal ids with cardiac reports regardless of status (e.g., Normal or Abnormal) that was downloaded from OFA. Information on each animal was then obtained by querying that animal’s full OFA report using the following standardized form: http://www.offa.org/display.html?regnum=[animal_ID]#animal where the animal ID was inserted into the ‘[animal_ID]’ portion of the URL. Information on the animal’s date of birth, fur color, sex, breed, registration number, test name, test date, report date (because the report date can differ from the test date) and test conclusion. This process resulted in a dataset of cats and dogs with cardiovascular report information. All cat breeds were excluded. The resulting dataset contained 129,783 canines with cardiovascular reports. Five of these contained only Null reports (i.e., no information on the test conclusion was provided). Therefore, these were excluded. The resulting dataset contained 179,585 canine cardiovascular reports from 129,778 unique canines representing 253 distinct dog breeds. Each of these canines included in our analysis had at least one publically accessible non-null cardiovascular report.

### Algorithm to Determine Canine Cardiovascular Status from OFA Reports

The OFA database provides information per dog on the registry type (e.g., cardiac, hips, elbow) and the final conclusion. We extracted all OFA reports for dogs having a ‘cardiac’ test. There are three ‘final conclusion’ choices that are made available on the OFA browser, including ‘abnormal’, ‘equivocal’, and ‘normal’. A veterinarian makes the designation of the dogs cardiovascular state based on the results of one or two tests, including Auscultation (i.e., listening with a stethoscope to the heart) and Electrocardiogram (ECG). The veterinarian then fills out the exam report and makes the ‘final conclusion’ regarding each dog’s cardiovascular state. The ECGs are not provided by the OFA and therefore, we were only provided with the final conclusion result.

**Figure 1:**
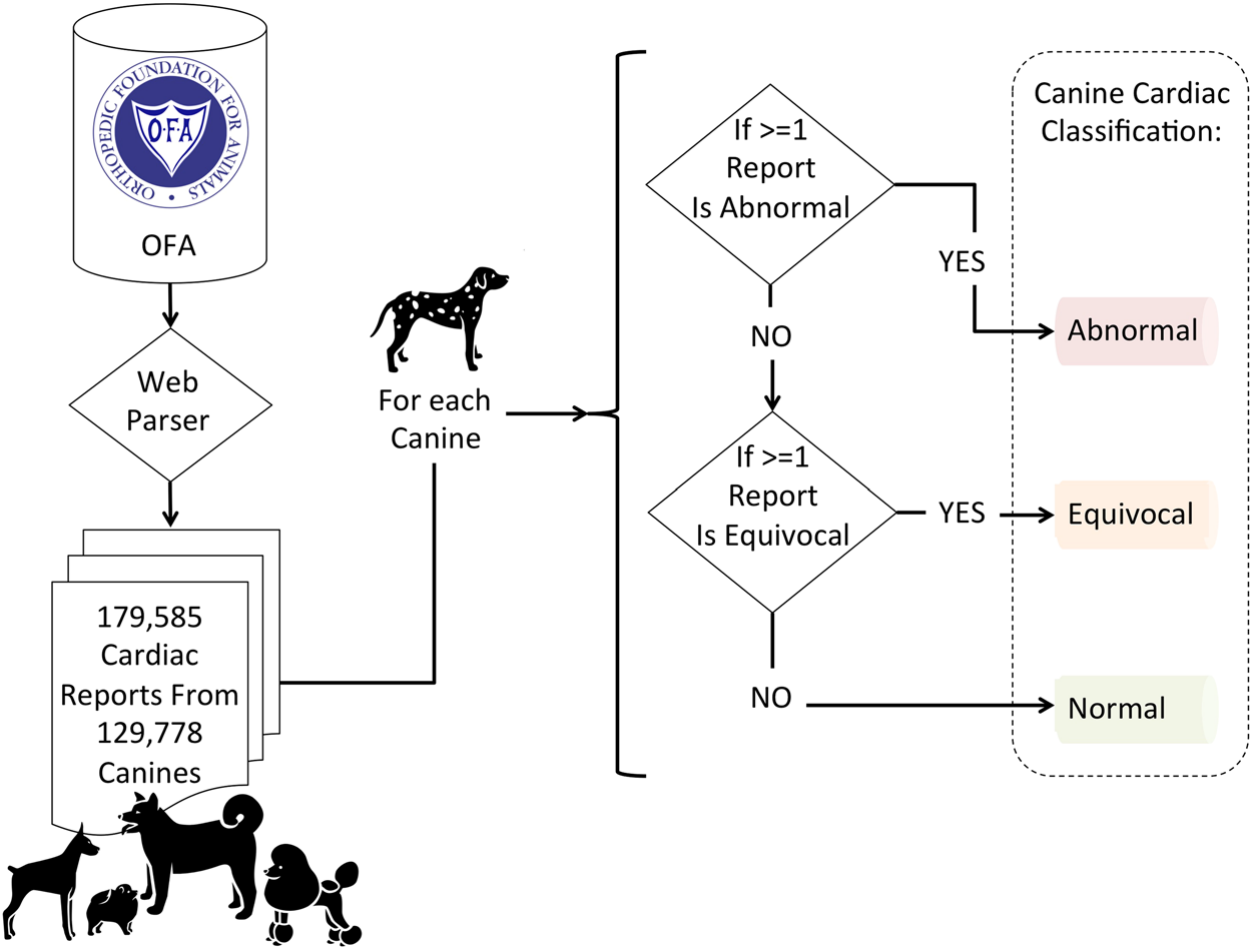
Algorithm for Processing OFA Cardiac Reports from Canines and Categorizing Each Canine’s Overall Cardiac Health Status. All dog icons (“akita”, “pomeranian”, “doberman”, “dalmatian”, “poodle”) within the figure are by: parkjisun, from thenounproject.com.

Because dogs can have more than one report listed in the OFA database, we build a decision tree algorithm (Fig. 1) to process the reports so that each dog was assigned as belonging to one of three cardiovascular health categories: abnormal, equivocal or normal. This was especially important for dogs with conflicting reports (e.g., one reporting ‘equivocal’ and one reporting ‘abnormal’). If any reports listed the canine as having an abnormal heart then the canine’s cardiac condition was listed as abnormal. If no abnormal reports exist and at least 1 report notes the heart as equivocal then the canine’s cardiac condition was listed as equivocal. Lastly, if no reports showed the heart as abnormal or equivocal (i.e., all reports listed the cardiovascular condition as being normal) then the canine was listed as being of normal cardiac condition.

### Integrating Breed and Breed Clade Information

Certain canine breeds have been associated with increased cardiovascular risk while others show decreased risk^9^. Therefore, in order to appropriately investigate a birth season – cardiovascular disease effect in canines it is important to categorize the dog breed effect. A 2017 paper by Parker *et al*. classified dog breeds into 23 distinct clades based on their genetic profiles^21^. A clade is a grouping of breeds based on genetic similarity, therefore several related breeds belong to the same clade^21^. For example, breeds like the Belgian malinois and German Shepherd would belong to the same clade while the Irish setter and the English setter would belong to another clade. We extracted the dog breed to clade information that was provided in the manuscript (Fig. 1 from their paper) to map the OFA dog breeds to clades. Of the 129,778 unique canines in our dataset, 116,655 matched to a known clade from the Parker *et al*.^21^ paper. We placed the remaining 13,123 dogs into an unknown clade. Parker *et al*. also had an unknown clade listed, but we created our own unknown category given that we investigated more distinct dog breeds (253 vs. 161 breeds) and therefore had more dog breeds of unknown clade.

Unfortunately, the Parker *et al*.^21^ study did not report cardiovascular disease risk by breed clade. Only overall genetic similarity was assessed. Therefore, we utilized a dataset from a literature review - Parker *et al*.^22^ - reporting the cardiovascular disease risk by dog breed for 13 distinct cardiovascular diseases (Fig. 1 from that paper)^22^. This allowed us to bin dogs into those with no known breed risk for any cardiovascular diseases and those with some breed risk for cardiovascular disease. We were also able to bin dogs into those at risk and those not at risk (due to their breed) for each of 13 common cardiovascular diseases^22^. Therefore, in addition to having breed clade information as a crude genetic relationship measure from Parker *et al*.^21^, we also had information on breed-cardiovascular disease risk from the Parker *et al*. 2006 study^22^.

### Regression Model for Investigating the Birth Month Effect

To study the relationship between birth month and cardiovascular disease risk among canines, we constructed a logistic regression model. The model predicts each canine’s risk of cardiovascular disease given a set of predictors. The outcome variable is binary (i.e., has cardiovascular disease or has no cardiovascular disease). We previously assigned canines as belonging to one of three classes (abnormal, equivocal or normal, see Fig. 1). For the purposes of this binary model, we grouped abnormal and equivocal together since both represent some type of cardiovascular abnormality either subtle or overt. Normal canines are those with no cardiovascular reports of either abnormal of equivocal findings. Birth month is modeled as a numeric variable (1–12 representing Jan–Dec); a binary variable represents the breeds known association with any cardiovascular diseases from Parker *et al*.^22^; and a categorical variable represents the breeds clade as reported in Parker *et al*.^21^. The unknown clade in our analysis includes not only breeds not studied and unclassifiable in Parker *et al*.^21^, but also hybrids, mutts and other non-breeds (e.g., ‘Labradoodle’). Hybrids, mixed-breeds or mutts are often thought to have lower cardiovascular disease risk due to hybrid vigor^23^, which makes them important to analyze. Other studies have shown mixed-breeds to be at higher risk of obesity, which may increase their risk of cardiovascular disease^24^.

### Relative Risk

Our regression model computes an adjusted odds ratio (OR), which is the odds that birth month affects risk of cardiovascular disease after accounting for the dog’s genetic predisposition for cardiovascular disease (i.e., from a breed known to be at risk for cardiovascular disease or not) and the overall breed effect, using the dog’s clade (i.e., a higher-level assessment of breed). Adjusted ORs can sometimes be misleading as they take into account all of these aforementioned confounding variables. Therefore, we also computed the Relative Risk (RR) of developing cardiovascular disease by birth month for dogs predisposed to cardiovascular disease and those not predisposed to cardiovascular disease. We used the raw proportions computed in the previous step to derive a RR.

1 $$\begin{array}{c}Relative\,Risk\,=\,\frac{The\,Absolute\,\,Risk\,of\,Events\,in\,the\,Treatment\,Group\,\,(ABT)}{The\,Absolute\,Risk\,of\,Events\,in\,the\,Control\,Group\,(ABC)}\\ \,\,\,\,\,\,\,=\,\frac{ABT}{\frac{Total\,Number\,with\,Cardiovascular\,Disease}{Total\,Number\,of\,Cardiovascular\,Disease\,Predisposed\,Dogs}}\end{array}$$

where

$$ABT\,=\,\frac{Cardiovascular\,Predisposed\,Dogs\,with\,Cardiovascular\,Disease\,By\,Birth\,Month}{Total\,Cardiovascular\,Predisposed\,Dogs\,By\,Birth\,Month}$$

The formula^25^ shown in Equation 1, allows us to compute a RR by birth month for dogs predisposed to cardiovascular disease. For our purposes, the Absolute risk of Events in the Control group (ABC) is the proportion of cardiovascular disease in the control group is the baseline incidence of cardiovascular disease in the dataset. Therefore, we did not use a particular birth month as our ‘control’ birth month; instead we used the baseline incidence as our control. The baseline incidence for cardiovascular disease (ABC) was computed separately for dogs predisposed to cardiovascular and those not predisposed to cardiovascular disease.

For the proportion of cardiovascular disease in the ‘treated’ group or ABT, this was the proportion of cardiovascular disease incidence by birth month (i.e., the proportion of those born in a given birth month – for example January – with cardiovascular disease out of those born in January). Likewise, we computed RR for cardiovascular disease by birth month also for dogs not predisposed to cardiovascular disease using the same formula except substituting cardiovascular predisposed dogs in Equation 1 with non-cardiovascular predisposed dogs.

## Results

### OFA Dataset and Breed Clade Information

**Figure 2:**
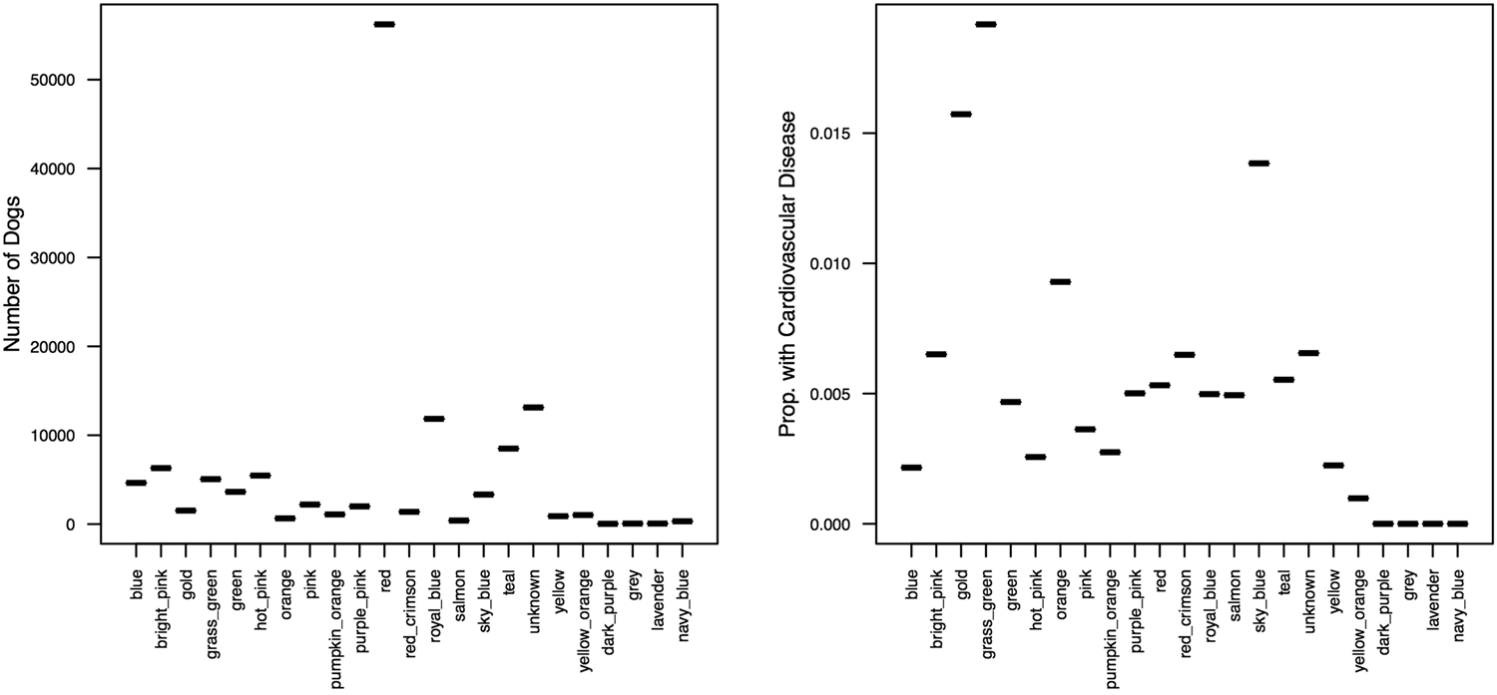
Prevalence of Breed Clade in the Public OFA Dataset (left-graph) and Proportion with Cardiovascular Disease per Breed Clade (right-graph). Some breeds (e.g., retrievers/pointers) were very common (left-graph red clade) while having only moderate levels of cardiovascular disease risk (right-graph red clade). While other clades (e.g., grass-green clade) had a high-risk of cardiovascular disease (right graph).

Our dataset contained 129,778 unique canines belonging to 23 distinct Parker clades (we had one clade as a catch-all clade for mixed breeds and other unclassified dog breeds from the Parker study). Of our canines, 10.11% belonged to this ‘unknown’ mixed-breed clade. We labeled each clade by the colors used in the Parker *et al*. paper, as they did not provide clade nomenclature^21^. For popular clades, we provide subtitles that represent the types of breeds belonging to each clade, see Fig. 2 and Table 1. The most popular clade was the retrievers and pointers (i.e., the red clade) with 56,224 canines representing 43.32% of the dataset. Prevalence of each breed clade is shown in Table 1 for clades with at least 1% prevalence in the OFA dataset. The percent t with cardiovascular disease within each clade is also reported in Table 1 with percent calculated as the number with cardiovascular disease/total canines in the clade.

**Table 1:** Prevalence of Breed Clades (for those with > = 1% Prevalence) in OFA Dataset and Incidence of Cardiovascular Disease.

Clade	Example Breeds within Clade	Prevalence of Breed Clade	Percent with Cardiovascular Disease
Red	Retrievers/Pointers	43.32%	0.53%
Unknown	Mixed Breeds, Rare Breeds	10.11%	0.66%
Royal Blue	Mastiffs/Bulldogs	9.13%	0.50%
Teal	Doberman Pinscher, Schnauzer, Rottweiler	6.55%	0.55%
Bright Pink	Great Dane, Rhodesian Ridgeback	4.85%	0.65%
Hot Pink	Maltese, Bichon Frise	4.21%	0.26%
Grass Green	Hound/Collie/Sheepdog	3.90%	1.92%
Blue	Saint Bernard, Bernese Mountain Dog, Greater Swiss Mountain Dog	2.80%	0.22%
Green	Belgian Malinois	2.56%	0.47%
Sky Blue	Terriers	1.70%	1.38%
Pink	Pomeranian/Pug/Papillon	1.54%	0.36%
Purple Pink	Chihuahua, Rat Terrier, American Hairless Terrier	1.18%	0.50%
Gold	German Shepherd	1.07%	1.57%

The prevalence of mixed-breeds and other breeds not classified into a genetic clade was low when compared against other studies. We report 10.11% of canines falling into this classification compared to 27% of canines being reported as mixed-breed from a large >30 k canine veterinary practice dataset^26^. This is most likely because our data comes from the OFA where primarily breeders submit their dogs’ data. This is because breeders seek to ensure the health of their sires and dams, along with the resulting offspring. However, typical pet owners are less likely to submit their dog’s results. This bias shifts the population in the OFA dataset towards canines of a known pure breed classification versus mixed breeds.

When we binned the data into breeds that are known to be at risk for cardiovascular disease^22^, we found that 92,057 canines were from breeds predisposed to cardiovascular disease compared to 37,721 canines from undisposed (or low cardiac risk) breeds. Therefore, 70.93% of OFA canines come from high-risk breeds and therefore are predisposed to cardiovascular disease.

**Figure 3:**
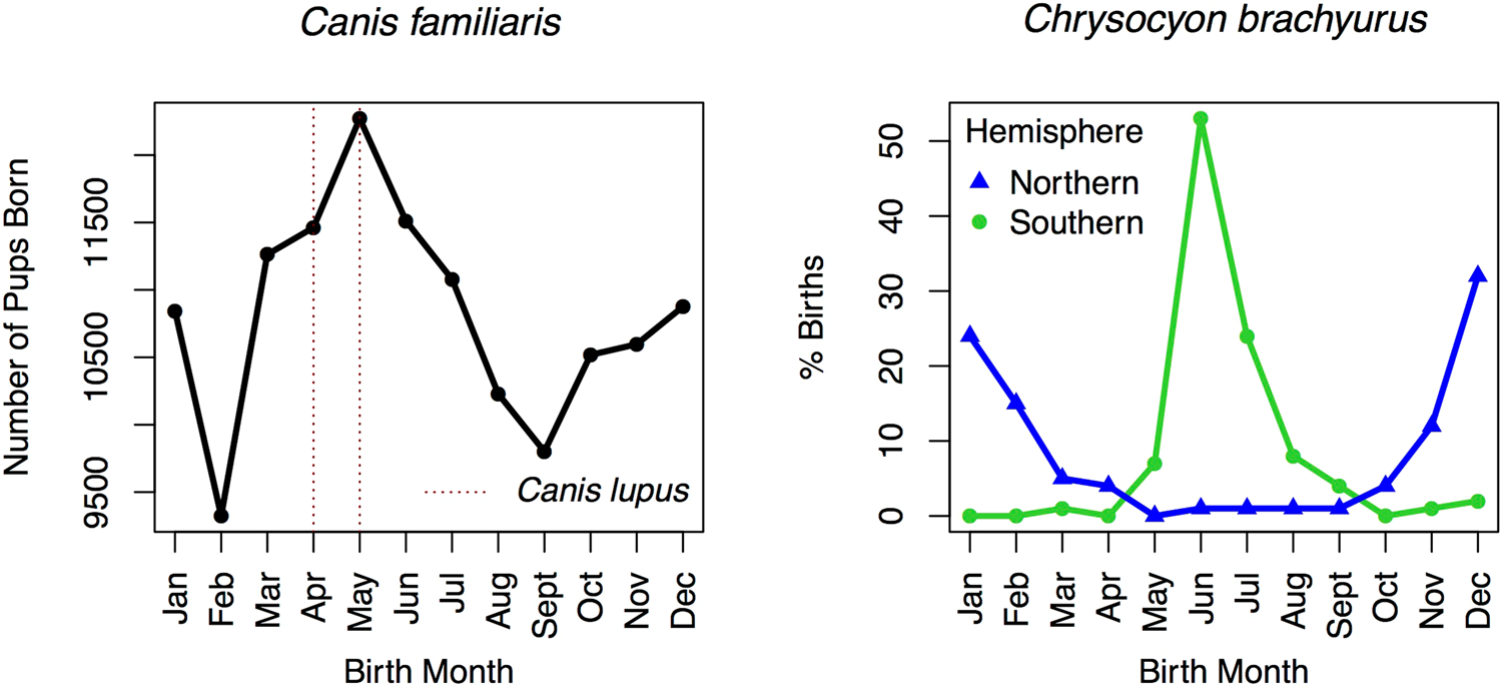
Number of *Canis familiaris* pups born per month in the OFA dataset (left-hand graph) and *Chrysocyon brachyurus* (maned wolf) born in captivity when exposed to Northern or Southern Hemisphere Climate. Left-hand graph shows the crude background birth rate by month of all pups/dogs included in the OFA dataset analyzed in this study. April and May are highlighted as these represent wild birth month dates for *Canis lupus* in extreme Northern climates^19^. Right-hand graph shows data on captive maned wolves (*Chrysocyon brachyurus*) left to breed in Northern or Southern Hemispheres from Maia et Gouveia^32^.

We plotted the background birth rate of the 129,778 unique canines from the OFA dataset included in our study (Fig. 3**)** illustrating a peak of births in May with March – July being the peak birth period for domesticated dogs. We highlight (dashed red lines in Fig. 3) April and May as these are the birth months for wild *Canis lupus* (wolf) when exposed to extreme Northern climates^19^. We also show the birth rate for captive maned wolves, or *Chrysocyon brachyurus*, that are indigenous to South America (right-hand graph in Fig. 3). Those born in the Southern Hemisphere show peak births in June and July while those born in the Northern Hemisphere (i.e., not the extreme Northern areas such as Alaska) show peaks in December–January. This helps to illustrate the differences between dogs from the OFA dataset, such as *Canis familiaris*, captive populations, such as *Chrysocyon brachyurus*, and wild populations, such as *Canis lupus*. There may be breed-specific effects that regulate the birth season process, however domestication is also a major factor in determining the birth season in the domesticated dog (left-hand graph in Fig. 3). Importantly, the peak birth month for domesticated dogs (May) differs from the peak birth month observed in humans in the USA (September)^10^.

### Regression Model for Investigating the Birth Month Effect

We constructed a regression model with a binary outcome variable for cardiovascular disease (i.e., abnormal or equivocal) versus healthy (i.e., normal). We decided to group abnormal and equivocal because the number of canines belonging to those classifications was low. There were 128,997 canines listed as normal, 428 as abnormal and 353 as equivocal. Our binary model binned abnormal and equivocal together for a total of 781 affected canines. We also modeled several confounder variables including breed clade from Parker *et al*.^21^, and whether or not the breed was predisposed to cardiovascular disease from Parker *et al*.^22^. We also added a variable for birth month, which was modeled as a numerical variable similar to other work performed in humans^10^. Two separate models were constructed. One included all 129,778 canines and the other model only contained the 116,655 canines having known breed clade information. In the restricted model, all mixed breeds and other rare uncharacterized breeds were excluded. Results are shown in Table 2. We also performed a chi-square test on the estimates from the model to obtain on overall breed clade p-value, which was <0.001 for both models shown in Table 2.

**Table 2:** Logistic Regression Model Results for Birth Month Effect on Cardiovascular Disease Incidence in Canines: Two Models.

Variable	All Canines (N = 129,778)		Breeds of Unknown Clade Excluded (e.g., Mixed-breeds) (N = 116,655)	
	OR (95% CI)	P-value	OR (95% CI)	P-value
Birth Month	1.02 (0.998, 1.040)	0.075	**1.02 (1.002, 1.047)**	**0.032**
Breed Clade	Varies	Varies*	Varies	Varies*
Cardiovascular Disease Predisposition Based On Breed	0.72	0.001	0.85	0.148

*A chi-square test on the estimates from the model obtained an overall breed clade p-value, p-value < 0.001 for both models.

The effect of birth month (as a proxy for birth season) on cardiovascular disease risk does not change between the two models and shows a steady increase in risk with an Odds Ratio (OR) of 1.02. Dogs belonging to a breed that is known to be at risk for cardiovascular disease are at lower risk of developing cardiovascular disease. This suggests the possibility of a selective breeding effect whereby breeds at risk for cardiovascular disease have screening programs in place to prevent affected dogs from reproducing. However, the significance of this protective effect also varies between the two models depending on whether or not the mixed-breed dogs (and other dogs of unknown clade) are included. Importantly, in both cases the effects were the same size and/or in the same direction (protective versus associated with increased disease risk). The p-value itself is sensitive to the sample size and the heterogeneity of the dogs included. Birth month was significant if dogs of unknown breed clade are excluded (see Discussion section). Also the overall effect of the breed clade was significant in both models. However, the individual breed clades that were significant and the strength of the associations varied slightly depending on the breed.

**Figure 4:**
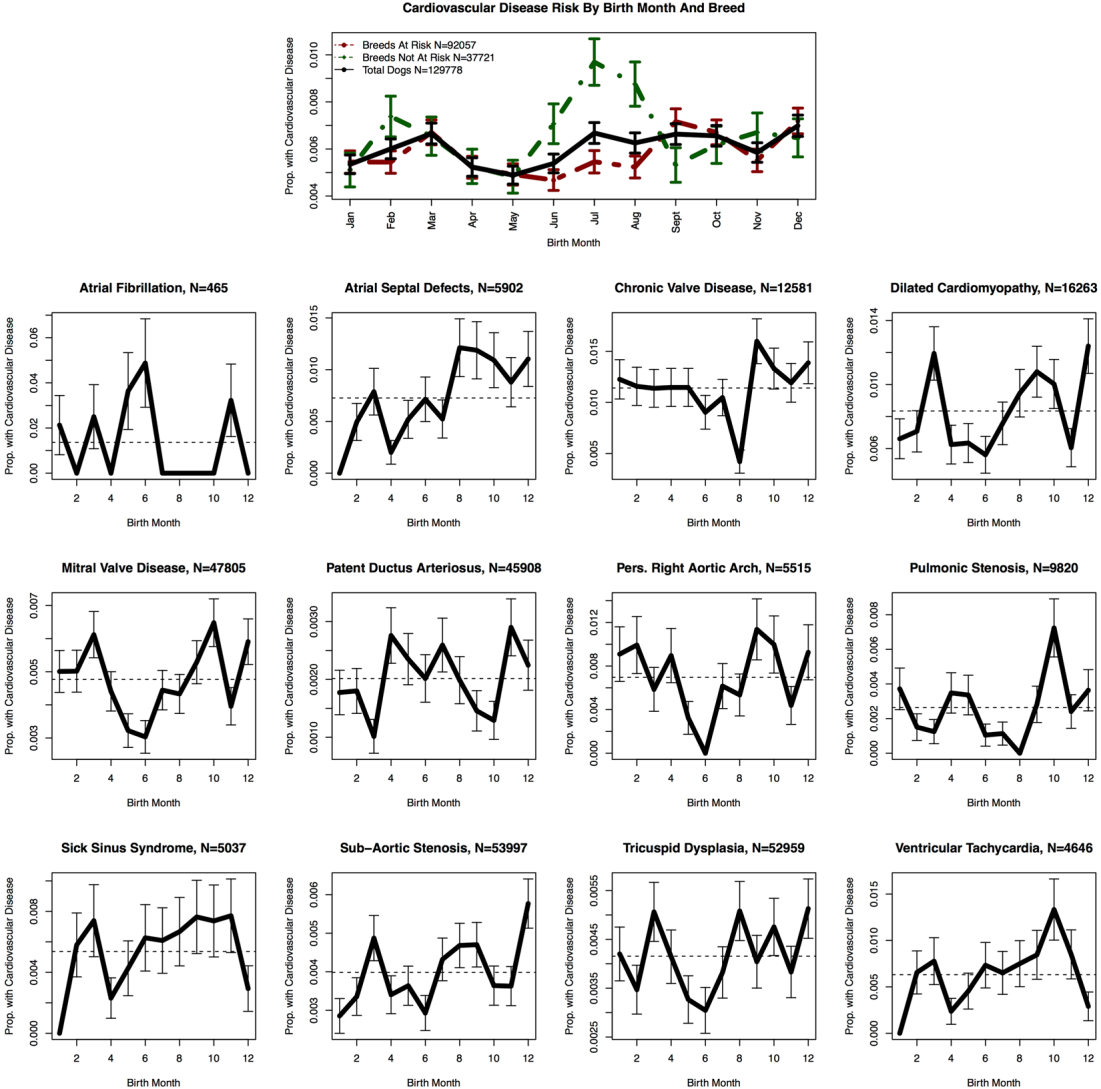
Cardiovascular Disease Risk Varies by Birth Month Among Canines that are not Predisposed to Cardiovascular Disease. The twelve subplots in the lower portion of the figure show the break down by birth month of dogs that are susceptible to that particular subtype of cardiovascular disease. The y-axis displays the proportion within those cohorts that developed cardiovascular disease.

There is a clear relationship between birth season and risk of cardiovascular disease among dog breeds that are NOT predisposed to cardiovascular disease (Fig. 4, top center graph**)**. The risk of cardiovascular disease among dogs born in the summer months (Jun-Aug) is greater for breeds not predisposed to cardiovascular disease (P = 0.038) versus those that are predisposed to cardiovascular disease (depicted in red in Fig. 4). We also include the overall cardiovascular disease risk by birth month for all dogs combined (black line in top center graph of Fig. 4).

We also investigated the birth season relationship among breeds pre-disposed to thirteen different cardiovascular diseases as reported in Parker *et al*.^22^. Some groups were low, e.g., only a few breeds have been implicated in increased risk for atrial fibrillation – accounting for only 465 canines in our population. Therefore, we were not able to detect a signal in those graphs. Breeds known to be susceptible to other cardiovascular diseases showed decreased risk among the summer months, including persistent right aortic arch; tricuspid dysplasia; and mitral valve disease. Atrial septal defects tended to increase towards the end of the year with peak months including August-December. Overall, the birth season – cardiovascular disease effect does appear to be modulated by the breed effect and also whether or not that particular breed is predisposed to cardiovascular disease. This is suggestive of a possible gene-environment interaction.

### Relative Risk of Cardiovascular Disease by Birth Month: Predisposed vs. Non-Predisposed

**Figure 5:**
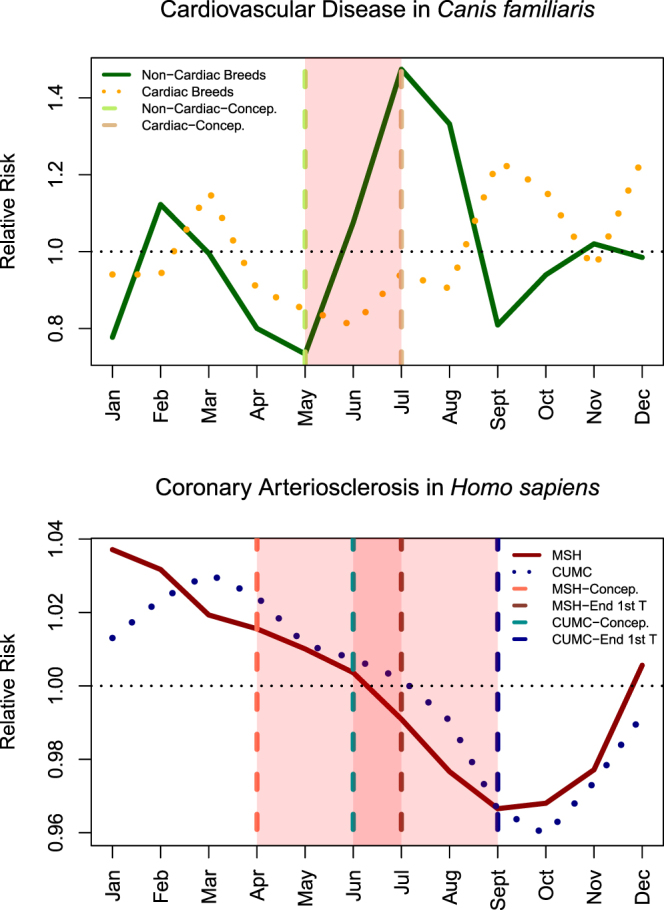
Cardiovascular Disease Risk By Birth Month in Canines (*Canis familiaris*) and Humans (*Homo sapiens*). The vertical lines represent the inferred conception month for the high-risk birth month in each group. Canines from breeds at risk for cardiovascular disease have a peak birth month in September, which corresponds to a July conception month. Canines from breeds not at risk for cardiovascular disease have a peak birth month in July corresponding to a May conception month. Shaded in red is the period between the two conception months. In humans (lower graph) there is some differences between Mount Sinai Hospital (MSH) and Columbia University Medical Center (CUMC). Again the inferred conception month for the peak birth month for cardiovascular risk is highlighted (dashed vertical lines, April for MSH and June for CUMC). The first trimester period for both MSH and CUMC for the peak coronary arteriosclerosis birth month at both sites (Jan – MSH, Mar – CUMC) is highlighted in red. We highlight the first trimester in humans, as this is the developmental period that is most comparable with canines. Notice the overlap between the highlighted red regions between canines (upper graph) and humans (lower graph) with regards to peak cardiovascular disease risk conception periods. There is overlap between these regions for June and July.

We computed the RR of developing cardiovascular disease by birth month for dogs predisposed to cardiovascular and those not predisposed (Fig. 5). The RR among dogs lacking a genetic predisposition to cardiovascular disease peaked at 1.47 for those dogs born in July. However, among dogs with a genetic predisposition the effect was weaker (highest RR was 1.24 observed among those born in both September and December, with September highlighted in Fig. 5). The RRs for each birth month for dogs predisposed to cardiovascular disease and those not predisposed to cardiovascular disease are shown in Table 3.

**Table 3:** Relative Risk for Cardiovascular Disease By Birth Month Among Canines Predisposed to Cardiovascular Disease and Those Not Predisposed to Cardiovascular Disease.

Birth Month	Relative Risk (RR) for Canines Predisposed to Cardiovascular Disease	Relative Risk (RR) for Canines Not Predisposed to Cardiovascular Disease
January	0.94	0.78
February	0.94	1.12
March	1.16	1
April	0.9	0.8
May	0.85	0.73
June	0.81	1.08
July	0.94	1.47
August	0.9	1.33
September	1.24	0.81
October	1.16	0.94
November	0.95	1.02
December	1.24	0.98

The overall OR for birth month – cardiovascular disease risk was 1.02 in the adjusted model meaning after breed clade and genetic predisposition for cardiovascular disease had been accounted for in the model (Table 2). Importantly, genetic predisposition was only important in the model that included mixed breeds (P = 0.001), but became insignificant when mixed-breeds were excluded (p = 0.148). Therefore, among purebred dogs (where we have greater power because the majority of submissions to the OFA are from breeders), the effect of genetic predisposition on cardiovascular disease was insignificant (p = 0.148) while the effect of birth month was significant (p = 0.032). This is important because it suggests that birth month is more informative on whether a dog will develop cardiovascular disease among purebred dogs in the OFA then genetic predisposition (Table 2).

### Comparison Between Humans and Canines: What Can We Learn?

We observed peak cardiovascular disease risk occurring for dogs born in July for dogs from breeds not at risk for cardiovascular disease and a peak in September for dogs from breeds at risk for cardiovascular disease (Fig. 5). Dogs born in the peak risk month had twice the risk of developing cardiovascular disease versus dogs born in the trough month (i.e., lowest risk month). Interestingly, the peak birth month for canines differs from our human studies^10,11^, which found peak cardiovascular disease risk occurring between January–April in New York City^10,11^. We calculated the inferred conception months and highlighted those in Fig. 5; we also highlighted in shaded red the region between the two inferred conception months in each subplot. Therefore, the shaded region for the canine subplot corresponds to when most dogs at risk for cardiovascular disease are conceived regardless of breed type (i.e., at risk or not at risk). For humans, we had two different sites in NYC and therefore the peak birth month varied from January at Mount Sinai Hospital (MSH) to April at Columbia University Medical Center (CUMC). This corresponds to a conception month of April (MSH) or August (CUMC). The shaded region is the peak conception region for births resulting in increased cardiovascular risk (Fig. 5).

## Discussion

### Acquired Cardiovascular Disease in Canines Demonstrates a Birth Season Effect

Cardiovascular disease, among dog breeds that are not predisposed to cardiac problems, appears to have a strong seasonal component as evidenced by the increase in cardiovascular disease among summer births (i.e., dark green line in Fig. 4). Because these dogs were not predisposed to cardiovascular disease then it is likely that this is evidence of an environmental effect, which does not appear to affect dogs belonging to breeds that are predisposed to cardiovascular disease. There are a couple of possible explanations for why this may be the case. The first is due to selective breeding. Dogs that are predisposed to cardiovascular disease are often monitored more than breeds that have no apparent risk of disease. For example, all Cavalier King Charles Spaniels are considered to have at least Class A cardiovascular disease even if no murmur is present^27^. In addition, dogs that show signs of cardiovascular disease that come from high-risk breeds are often prevented from breeding^27^ along with other disease-related conditions^28^. This prevents them from passing on their genes to the future generation thereby improving the stock quality of the breed. However, dogs belonging to breeds that are not at increased risk of cardiovascular disease may not undergo as stringent testing for cardiovascular disease.

Congenital cardiovascular disease is often easily detected when dogs are still puppies. This allows breeders to make decisions regarding which puppies to be kept for breeding purposes and which should be neutered/spayed and thus sold as pets. Importantly inheritance of cardiovascular disease, even among predisposed breeds, does not follow a Mendelian inheritance pattern^29^. Suggestive of other factors involved in the development of cardiovascular disease, beyond simply genetics.

Acquired cardiovascular disease is more difficult to detect than the congenital form and often remains latent until later in life. In addition, acquired cardiovascular disease is thought to be due to a myriad of factors, including obesity, exercise, diet and also genetic factors. Because acquired cardiovascular disease is acquired later in life and is often less breed-specific, breeders have difficulty monitoring it as stringently as they do for congenital cardiovascular disease. The results of our study strongly suggest that acquired cardiovascular disease displays a birth-season component as evidenced by the increase in cardiovascular disease among summer births of dogs that were not predisposed to cardiovascular disease. Our findings appear similar to what was found in our previous human studies where acquired cardiovascular diseases (i.e., not congenital cardiovascular diseases) were birth month/season dependent^10,11^. However, the odds ratios are lower in this canine study, which could be due to the lack of region of birth information for the dogs included in our study. Lacking region of birth for our canines introduces noise because we are unable to distinguish those dogs born in southern regions (e.g., Texas) from those born in the north (e.g., Indiana). This noise in our dataset may mean that the effect size we observe may be lower then the true effect size.

### Gene-Environment Interactions Affecting the Birth Season Effect are Likely

The birth season effect among canines does not appear as pronounced among dogs that are predisposed to cardiovascular disease. Therefore, it is likely that a genetic characteristic of dogs not typically at risk for cardiovascular disease is enhancing those dogs’ susceptibility to an ‘adverse effect of seasonality’ (i.e., developing acquired cardiovascular disease and being born in a high-risk month). This also suggests the possibility that the gene involved in modulating this effect is not a typical gene thought to be involved in cardiovascular disease. If the gene were a traditional cardiovascular disease gene, we would most likely have observed a stronger signal among canines predisposed to cardiovascular disease. However, since the effect was the reverse, it is likely not a traditional cardiovascular disease gene that is modulating the birth season effect.

### Potential for Selection Bias Among Breeders of Dogs Predisposed to Cardiovascular Disease

We observed an increase of cardiovascular disease risk for dogs born in June-August that belong to breeds not known to be predisposed to cardiovascular disease. However, breeders are not required to submit data to the OFA that is publically available. They can register their dogs via a private mechanism. Dogs registered via the private mechanisms are not included in this study, which utilizes only publically available OFA data. Breeders decide whether or not they want to make their dogs’ data public and therefore there may be some selection biases in this respect. For example, it is possible that breeders of dogs that are known to be at risk for cardiovascular problems may choose not to publically share their dogs’ results. This reporting bias could affect the results in various ways and is the subject of future work. We mention this explicitly to make readers aware of this factor. We are encouraged that the birth season effect was observed in the dogs not predisposed to cardiovascular disease because we believe breeders of dogs not predisposed to cardiac problems may be less likely to block their dogs’ results from the public. However, more work is needed in assessing these biases and is the subject of our future work.

### What Can We Learn About the Exposure’s Mechanism and Gestational Effects from Canines?

We observe some overlap between the peak conception periods across breed types (non-cardiac risk and cardiac risk) in dogs (*Canis familiaris*) and sites in humans from NYC (*Homo sapiens*) as shown in Fig. 5. This suggests the possibility that the cardiovascular disease risk exposure may be closer to conception then previously thought (i.e., conception or first-trimester exposure). Interestingly, we performed a global analysis of birth season relationships using data from the United States of America (including NYC, Nashville Tennessee and Seattle Washington) along with several sites in Asia (including South Korea and Taiwan) and found that first trimester exposure to fine air particulates (PM 2.5) was the strongest correlate to explain the birth seasonal variance in increased risk of atrial fibrillation in humans^13^. This current analysis in dogs also seems to suggest a first trimester exposure being the main culprit behind the increased risk of cardiovascular disease by birth month.

Mapping developmental processes across species is challenging. Puppies are born with both their eyes shut and ear canals closed and they remain so for the first three weeks of life. This makes newborn puppies functionally blind and deaf. Therefore, when comparing developmental stages such as ‘first trimester’ in humans with a similar stage of development in canines is challenging. However, our results suggest that the environmental exposure that is driving the cardiovascular disease – birth month association is an association that occurs early on in human development and not a perinatal exposure. This fits well with the literature on human cardiovascular development, which has consistently demonstrated that first trimester exposure to certain chemicals increases the risk for structural anomalies of the heart^30,31^. More work is needed to explore what type of exposure may be at play and how the canine and hominoid hearts are affected during the course of development resulting in a latent effect of birth seasonality on the heart.

### Limitations and Future Work

There are several limitations of our work. The OFA dataset contains self-reported canine health data from breeders and owners and their test results for a variety of conditions, including cardiovascular conditions. This dataset is biased towards dogs that are used for breeding purposes (i.e., breeding stock) as breeders are required to test their animals and report the results to a variety of agencies. However, only those breeders who chose to provide their dogs’ data publically were included in this study, and this biases the sample (the OFA does have an option for owners/breeders to submit their dogs’ data without having the data appear on the public website). Therefore, it is highly likely that breeders who receive information that their dog has cardiovascular issues may elect to not provide this data publically. Also in general, since these dogs are breeding stock, they are likely to be healthier and from more affluent owners. Many pets are also included in the OFA dataset, however they are under-represented. This is evidenced by the low prevalence of mixed-breeds in our study (10% of dogs belonged to the mixed-breed clade) when compared to other studies that report close to 30% mixed-breed population. Additionally, we were unable to adjust for many other factors that influence cardiovascular disease risk because those variables were not available in the dataset. These include, but are not limited to: obesity, diet, exercise regime, and region of birth.

Another limitation of this study is that the time to cardiovascular diagnosis was not considered. In most cases, there was only one cardiovascular test per dog, but for dogs with multiple testing, we could consider the time to first detection of a cardiovascular condition or anomaly per dog. This is difficult using OFA data alone, as other cardiovascular functioning tests may have been performed that were not reported to the OFA. Therefore, this will be the subject of future, more detailed, work on subpopulations with more data.

Future work includes validating our canine cardiovascular disease – birth month associations at other sites and datasets to further probe this interesting relationship between breed, birth season and risk of cardiovascular disease. We also seek to explore further the role of breeder selection bias in the decision to report cardiovascular test results to the public (via the open-access mechanism). In addition, we hope to include some information from human studies on birth month to hone in on the exposure underlying this birth season – increased risk of cardiovascular disease relationship^13^. This will allow us to effectively add birth year to our analysis to determine if variance in the exposure (e.g., fine air particulates) by birth year affects the risk of cardiovascular disease.

## Conclusion

In conclusion, we performed a retrospective analysis of birth month’s effect on cardiovascular disease risk in canines. We specifically investigated a relationship between cardiovascular disease and birth month. We sought to investigate canines because of the physiological similarity between human and canine cardiovascular systems. Our analysis shows a clear birth season relationship among dogs belonging to breeds that are not predisposed to cardiovascular disease. This suggests that acquired cardiovascular disease may be birth season dependent among all dog breeds. The overall odds ratio for the birth season effect was 1.02 among all dogs while adjusting for breed and predisposition effects. The odds ratio observed in canines is on par with odds ratios reported in humans, which suggests that there may be a true birth season effect in cardiovascular disease. Our findings indicate that acquired cardiovascular disease among dogs, especially those that are not predisposed to cardiovascular disease, is birth season dependent. Studying birth season effects in model organisms can help to elucidate potential mechanisms behind the reported associations. We can also study birth season effects in canines to elucidate causal mechanisms underlying the birth season – cardiovascular disease relationship.
